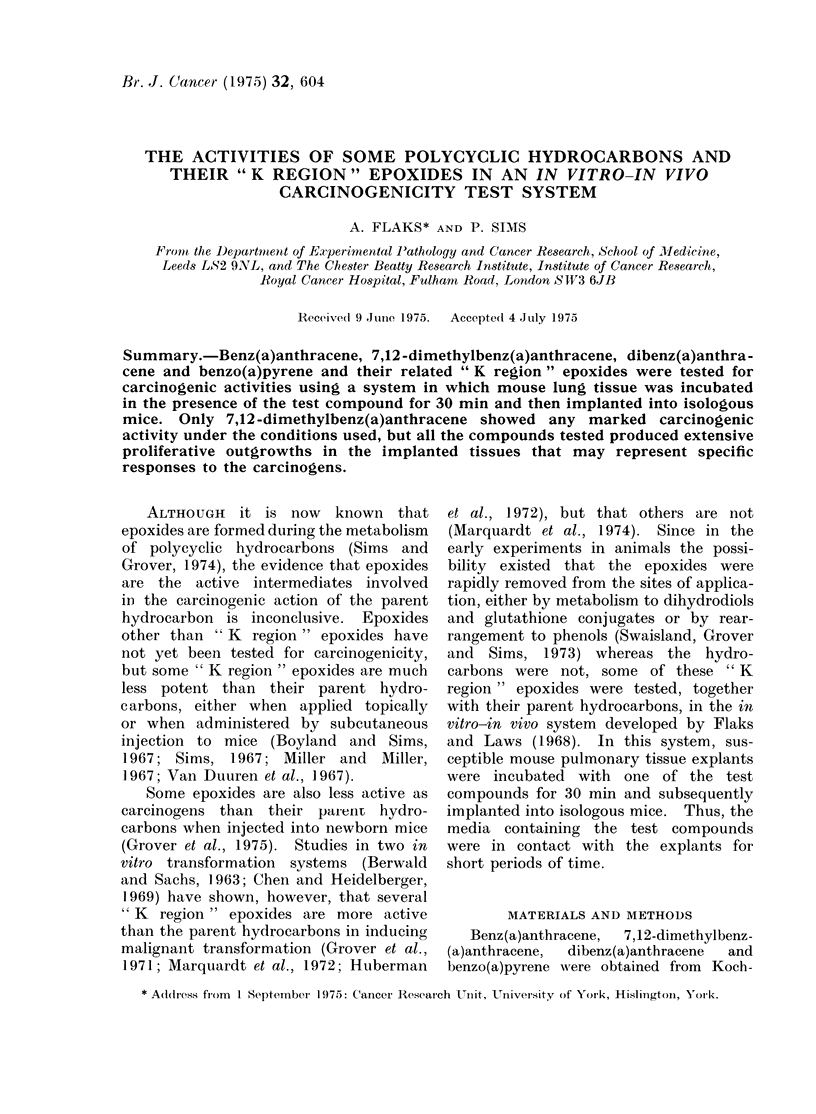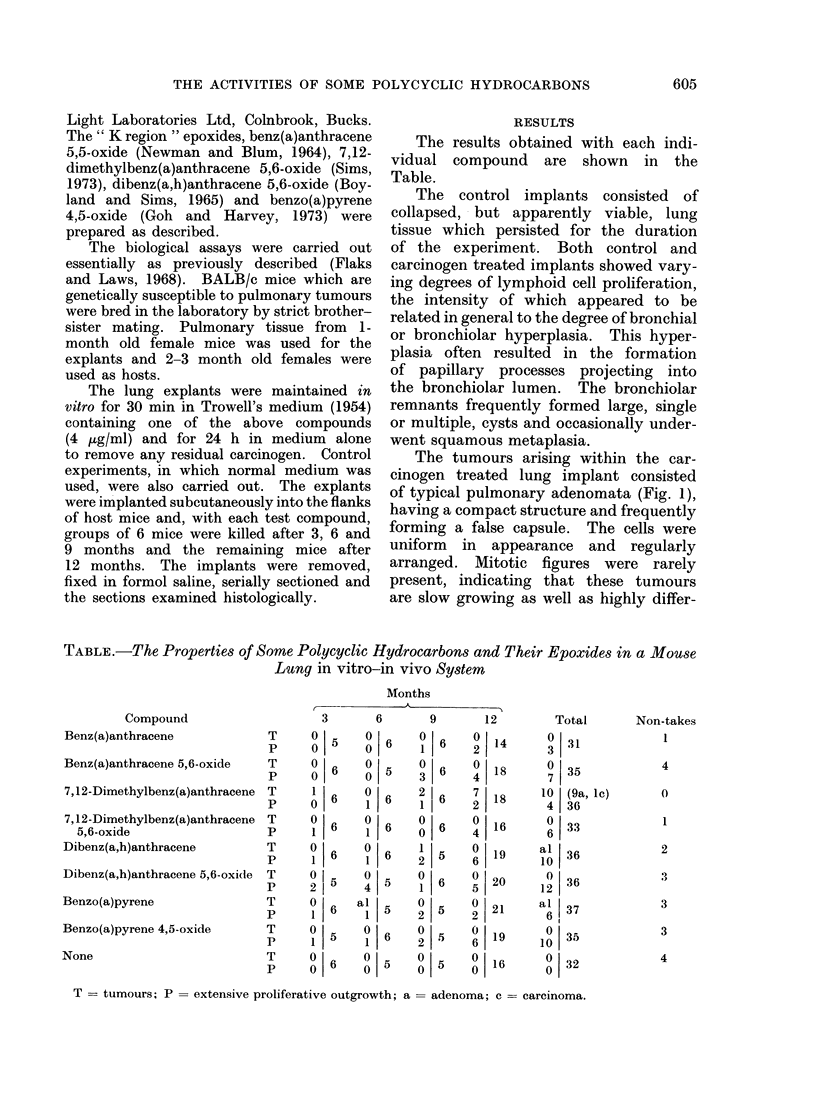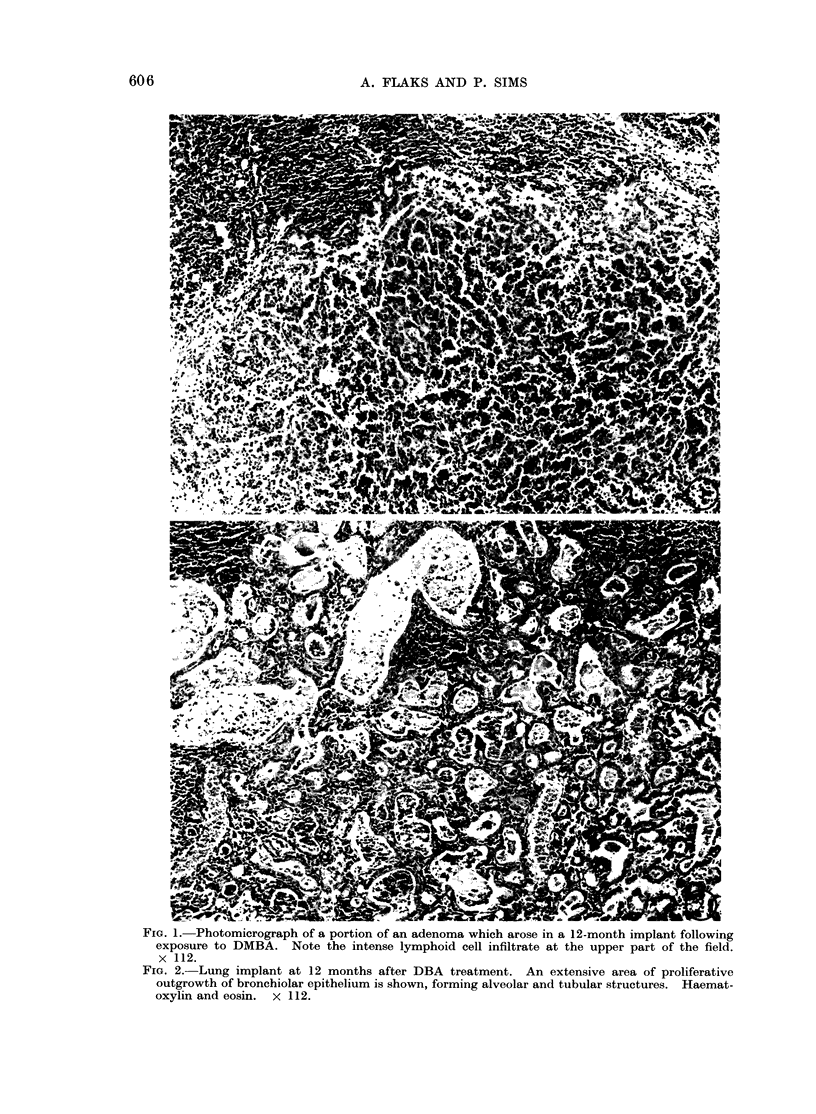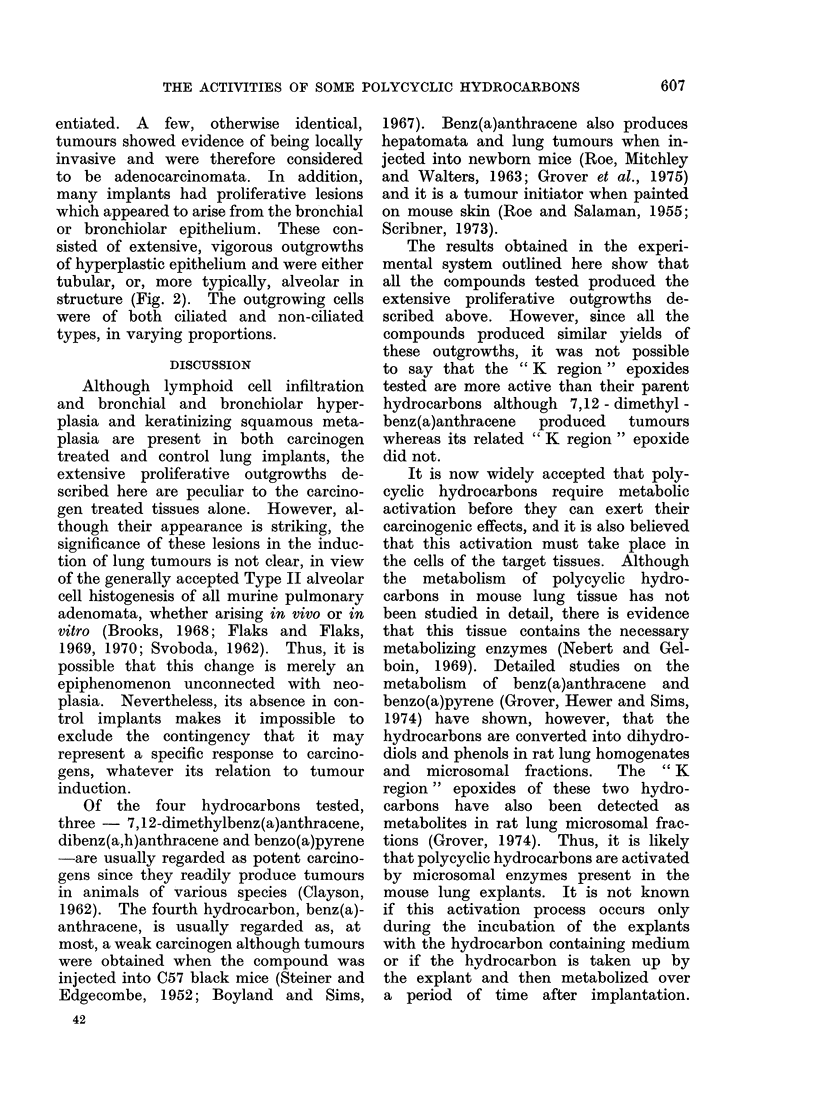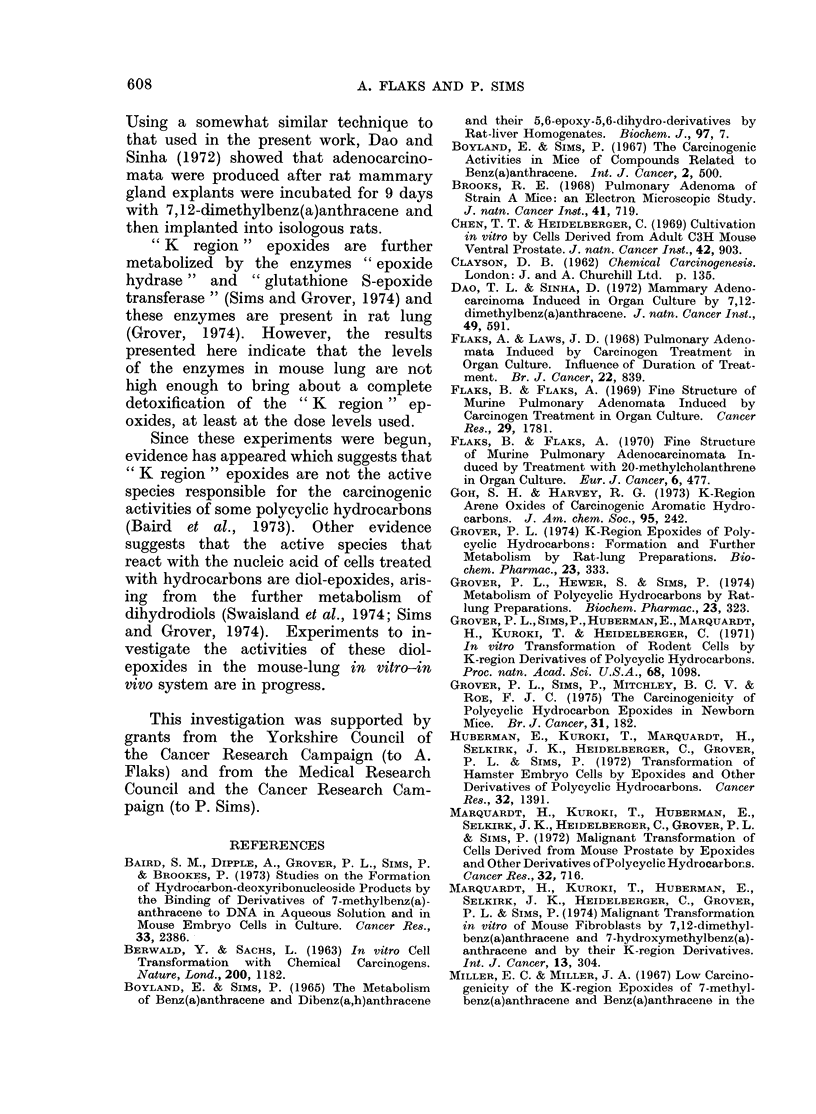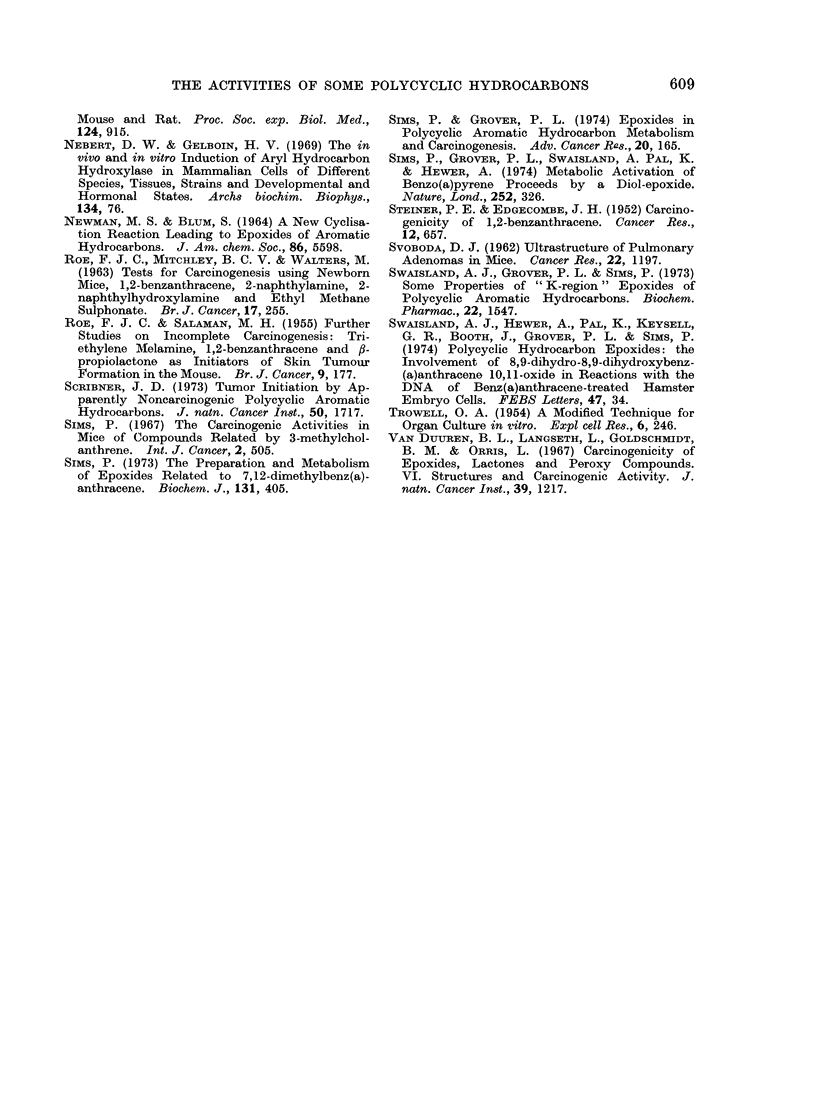# The activities of some polycyclic hydrocarbons and their "K region" epoxides in an in vitro-in vivo carcinogenicity test system.

**DOI:** 10.1038/bjc.1975.267

**Published:** 1975-11

**Authors:** A. Flaks, P. Sims

## Abstract

**Images:**


Br. J. Cancer (1975) **32**, 604

### THE ACTIVITIES OF SOME POLYCYCLIC HYDROCARBONS AND THEIR "K REGION" EPOXIDES IN AN *IN VITRO–IN VIVO* CARCINOGENICITY TEST SYSTEM

A. FLAKS* AND P. SIMS

*From the Department of Experimental Pathology and Cancer Research, School of Medicine, Leeds LS2 9NL, and The Chester Beatty Research Institute, Institute of Cancer Research, Royal Cancer Hospital, Fulham Road, London SW3 6JB*

Received 9 June 1975. Accepted 4 July 1975

**Summary.—Benz(a)anthracene, 7,12-dimethylbenz(a)anthracene, dibenz(a)anthracene and benzo(a)pyrene and their related "K region" epoxides were tested for carcinogenic activities using a system in which mouse lung tissue was incubated in the presence of the test compound for 30 min and then implanted into isologous mice. Only 7,12-dimethylbenz(a)anthracene showed any marked carcinogenic activity under the conditions used, but all the compounds tested produced extensive proliferative outgrowths in the implanted tissues that may represent specific responses to the carcinogens.**

ALTHOUGH it is now known that epoxides are formed during the metabolism of polycyclic hydrocarbons (Sims and Grover, 1974), the evidence that epoxides are the active intermediates involved in the carcinogenic action of the parent hydrocarbon is inconclusive. Epoxides other than "K region" epoxides have not yet been tested for carcinogenicity, but some "K region" epoxides are much less potent than their parent hydrocarbons, either when applied topically or when administered by subcutaneous injection to mice (Boyland and Sims, 1967; Sims, 1967; Miller and Miller, 1967; Van Duuren *et al.*, 1967).

Some epoxides are also less active as carcinogens than their parent hydrocarbons when injected into newborn mice (Grover *et al.*, 1975). Studies in two *in vitro* transformation systems (Berwald and Sachs, 1963; Chen and Heidelberger, 1969) have shown, however, that several "K region" epoxides are more active than the parent hydrocarbons in inducing malignant transformation (Grover *et al.*, 1971; Marquardt *et al.*, 1972; Huberman *et al.*, 1972), but that others are not (Marquardt *et al.*, 1974). Since in the early experiments in animals the possibility existed that the epoxides were rapidly removed from the sites of application, either by metabolism to dihydrodiols and glutathione conjugates or by rearrangement to phenols (Swaisland, Grover and Sims, 1973) whereas the hydrocarbons were not, some of these "K region" epoxides were tested, together with their parent hydrocarbons, in the *in vitro–in vivo* system developed by Flaks and Laws (1968). In this system, susceptible mouse pulmonary tissue explants were incubated with one of the test compounds for 30 min and subsequently implanted into isologous mice. Thus, the media containing the test compounds were in contact with the explants for short periods of time.

### MATERIALS AND METHODS

Benz(a)anthracene, 7,12-dimethylbenz(a)anthracene, dibenz(a)anthracene and benzo(a)pyrene were obtained from Koch-

* Address from 1 September 1975: Cancer Research Unit, University of York, Hislington, York.

Light Laboratories Ltd, Colnbrook, Bucks. The " K region " epoxides, benz(a)anthracene 5,5-oxide (Newman and Blum, 1964), 7,12-dimethylbenz(a)anthracene 5,6-oxide (Sims, 1973), dibenz(a,h)anthracene 5,6-oxide (Boyland and Sims, 1965) and benzo(a)pyrene 4,5-oxide (Goh and Harvey, 1973) were prepared as described.

The biological assays were carried out essentially as previously described (Flaks and Laws, 1968). BALB/c mice which are genetically susceptible to pulmonary tumours were bred in the laboratory by strict brother–sister mating. Pulmonary tissue from 1-month old female mice was used for the explants and 2–3 month old females were used as hosts.

The lung explants were maintained *in vitro* for 30 min in Trowell's medium (1954) containing one of the above compounds (4 μg/ml) and for 24 h in medium alone to remove any residual carcinogen. Control experiments, in which normal medium was used, were also carried out. The explants were implanted subcutaneously into the flanks of host mice and, with each test compound, groups of 6 mice were killed after 3, 6 and 9 months and the remaining mice after 12 months. The implants were removed, fixed in formol saline, serially sectioned and the sections examined histologically.

### RESULTS

The results obtained with each individual compound are shown in the Table.

The control implants consisted of collapsed, but apparently viable, lung tissue which persisted for the duration of the experiment. Both control and carcinogen treated implants showed varying degrees of lymphoid cell proliferation, the intensity of which appeared to be related in general to the degree of bronchial or bronchiolar hyperplasia. This hyperplasia often resulted in the formation of papillary processes projecting into the bronchiolar lumen. The bronchiolar remnants frequently formed large, single or multiple, cysts and occasionally underwent squamous metaplasia.

The tumours arising within the carcinogen treated lung implant consisted of typical pulmonary adenomata (Fig. 1), having a compact structure and frequently forming a false capsule. The cells were uniform in appearance and regularly arranged. Mitotic figures were rarely present, indicating that these tumours are slow growing as well as highly differ-

TABLE.—*The Properties of Some Polycyclic Hydrocarbons and Their Epoxides in a Mouse Lung* in vitro–in vivo *System*

| Compound | | Months 3 | | 6 | | 9 | | 12 | | Total | | Non-takes |
|---|---|---|---|---|---|---|---|---|---|---|---|---|
| Benz(a)anthracene | T | 0 | 5 | 0 | 6 | 0 | 6 | 0 | 14 | 0 | 31 | 1 |
| | P | 0 | | 0 | | 1 | | 2 | | 3 | | |
| Benz(a)anthracene 5,6-oxide | T | 0 | 6 | 0 | 5 | 0 | 6 | 0 | 18 | 0 | 35 | 4 |
| | P | 0 | | 0 | | 3 | | 4 | | 7 | | |
| 7,12-Dimethylbenz(a)anthracene | T | 1 | 6 | 0 | 6 | 2 | 6 | 7 | 18 | 10 | (9a, 1c) | 0 |
| | P | 0 | | 1 | | 1 | | 2 | | 4 | 36 | |
| 7,12-Dimethylbenz(a)anthracene | T | 0 | 6 | 0 | 6 | 0 | 6 | 0 | 16 | 0 | 33 | 1 |
| 5,6-oxide | P | 1 | | 1 | | 0 | | 4 | | 6 | | |
| Dibenz(a,h)anthracene | T | 0 | 6 | 0 | 6 | 1 | 5 | 0 | 19 | a1 | 36 | 2 |
| | P | 1 | | 1 | | 2 | | 6 | | 10 | | |
| Dibenz(a,h)anthracene 5,6-oxide | T | 0 | 5 | 0 | 5 | 0 | 6 | 0 | 20 | 0 | 36 | 3 |
| | P | 2 | | 4 | | 1 | | 5 | | 12 | | |
| Benzo(a)pyrene | T | 0 | 6 | a1 | 5 | 0 | 5 | 0 | 21 | a1 | 37 | 3 |
| | P | 1 | | 1 | | 2 | | 2 | | 6 | | |
| Benzo(a)pyrene 4,5-oxide | T | 0 | 5 | 0 | 6 | 0 | 5 | 0 | 19 | 0 | 35 | 3 |
| | P | 1 | | 1 | | 2 | | 6 | | 10 | | |
| None | T | 0 | 6 | 0 | 5 | 0 | 5 | 0 | 16 | 0 | 32 | 4 |
| | P | 0 | | 0 | | 0 | | 0 | | 0 | | |

T = tumours; P = extensive proliferative outgrowth; a = adenoma; c = carcinoma.

FIG. 1.—Photomicrograph of a portion of an adenoma which arose in a 12-month implant following exposure to DMBA. Note the intense lymphoid cell infiltrate at the upper part of the field. × 112.

FIG. 2.—Lung implant at 12 months after DBA treatment. An extensive area of proliferative outgrowth of bronchiolar epithelium is shown, forming alveolar and tubular structures. Haematoxylin and eosin. × 112.

entiated. A few, otherwise identical, tumours showed evidence of being locally invasive and were therefore considered to be adenocarcinomata. In addition, many implants had proliferative lesions which appeared to arise from the bronchial or bronchiolar epithelium. These consisted of extensive, vigorous outgrowths of hyperplastic epithelium and were either tubular, or, more typically, alveolar in structure (Fig. 2). The outgrowing cells were of both ciliated and non-ciliated types, in varying proportions.

### DISCUSSION

Although lymphoid cell infiltration and bronchial and bronchiolar hyperplasia and keratinizing squamous metaplasia are present in both carcinogen treated and control lung implants, the extensive proliferative outgrowths described here are peculiar to the carcinogen treated tissues alone. However, although their appearance is striking, the significance of these lesions in the induction of lung tumours is not clear, in view of the generally accepted Type II alveolar cell histogenesis of all murine pulmonary adenomata, whether arising *in vivo* or *in vitro* (Brooks, 1968; Flaks and Flaks, 1969, 1970; Svoboda, 1962). Thus, it is possible that this change is merely an epiphenomenon unconnected with neoplasia. Nevertheless, its absence in control implants makes it impossible to exclude the contingency that it may represent a specific response to carcinogens, whatever its relation to tumour induction.

Of the four hydrocarbons tested, three — 7,12-dimethylbenz(a)anthracene, dibenz(a,h)anthracene and benzo(a)pyrene —are usually regarded as potent carcinogens since they readily produce tumours in animals of various species (Clayson, 1962). The fourth hydrocarbon, benz(a)-anthracene, is usually regarded as, at most, a weak carcinogen although tumours were obtained when the compound was injected into C57 black mice (Steiner and Edgecombe, 1952; Boyland and Sims, 1967). Benz(a)anthracene also produces hepatomata and lung tumours when injected into newborn mice (Roe, Mitchley and Walters, 1963; Grover *et al.*, 1975) and it is a tumour initiator when painted on mouse skin (Roe and Salaman, 1955; Scribner, 1973).

The results obtained in the experimental system outlined here show that all the compounds tested produced the extensive proliferative outgrowths described above. However, since all the compounds produced similar yields of these outgrowths, it was not possible to say that the " K region " epoxides tested are more active than their parent hydrocarbons although 7,12 - dimethyl - benz(a)anthracene produced tumours whereas its related " K region " epoxide did not.

It is now widely accepted that polycyclic hydrocarbons require metabolic activation before they can exert their carcinogenic effects, and it is also believed that this activation must take place in the cells of the target tissues. Although the metabolism of polycyclic hydrocarbons in mouse lung tissue has not been studied in detail, there is evidence that this tissue contains the necessary metabolizing enzymes (Nebert and Gelboin, 1969). Detailed studies on the metabolism of benz(a)anthracene and benzo(a)pyrene (Grover, Hewer and Sims, 1974) have shown, however, that the hydrocarbons are converted into dihydrodiols and phenols in rat lung homogenates and microsomal fractions. The " K region " epoxides of these two hydrocarbons have also been detected as metabolites in rat lung microsomal fractions (Grover, 1974). Thus, it is likely that polycyclic hydrocarbons are activated by microsomal enzymes present in the mouse lung explants. It is not known if this activation process occurs only during the incubation of the explants with the hydrocarbon containing medium or if the hydrocarbon is taken up by the explant and then metabolized over a period of time after implantation.

Using a somewhat similar technique to that used in the present work, Dao and Sinha (1972) showed that adenocarcinomata were produced after rat mammary gland explants were incubated for 9 days with 7,12-dimethylbenz(a)anthracene and then implanted into isologous rats.

" K region " epoxides are further metabolized by the enzymes " epoxide hydrase " and " glutathione S-epoxide transferase " (Sims and Grover, 1974) and these enzymes are present in rat lung (Grover, 1974). However, the results presented here indicate that the levels of the enzymes in mouse lung are not high enough to bring about a complete detoxification of the " K region " epoxides, at least at the dose levels used.

Since these experiments were begun, evidence has appeared which suggests that " K region " epoxides are not the active species responsible for the carcinogenic activities of some polycyclic hydrocarbons (Baird *et al.*, 1973). Other evidence suggests that the active species that react with the nucleic acid of cells treated with hydrocarbons are diol-epoxides, arising from the further metabolism of dihydrodiols (Swaisland *et al.*, 1974; Sims and Grover, 1974). Experiments to investigate the activities of these diol-epoxides in the mouse-lung *in vitro–in vivo* system are in progress.

This investigation was supported by grants from the Yorkshire Council of the Cancer Research Campaign (to A. Flaks) and from the Medical Research Council and the Cancer Research Campaign (to P. Sims).

### REFERENCES

BAIRD, S. M., DIPPLE, A., GROVER, P. L., SIMS, P. & BROOKES, P. (1973) Studies on the Formation of Hydrocarbon-deoxyribonucleoside Products by the Binding of Derivatives of 7-methylbenz(a)-anthracene to DNA in Aqueous Solution and in Mouse Embryo Cells in Culture. *Cancer Res.*, **33**, 2386.

BERWALD, Y. & SACHS, L. (1963) *In vitro* Cell Transformation with Chemical Carcinogens. *Nature, Lond.*, **200**, 1182.

BOYLAND, E. & SIMS, P. (1965) The Metabolism of Benz(a)anthracene and Dibenz(a,h)anthracene and their 5,6-epoxy-5,6-dihydro-derivatives by Rat-liver Homogenates. *Biochem. J.*, **97**, 7.

BOYLAND, E. & SIMS, P. (1967) The Carcinogenic Activities in Mice of Compounds Related to Benz(a)anthracene. *Int. J. Cancer*, **2**, 500.

BROOKS, R. E. (1968) Pulmonary Adenoma of Strain A Mice: an Electron Microscopic Study. *J. natn. Cancer Inst.*, **41**, 719.

CHEN, T. T. & HEIDELBERGER, C. (1969) Cultivation *in vitro* by Cells Derived from Adult C3H Mouse Ventral Prostate. *J. natn. Cancer Inst.*, **42**, 903.

CLAYSON, D. B. (1962) *Chemical Carcinogenesis.* London: J. and A. Churchill Ltd. p. 135.

DAO, T. L. & SINHA, D. (1972) Mammary Adenocarcinoma Induced in Organ Culture by 7,12-dimethylbenz(a)anthracene. *J. natn. Cancer Inst.*, **49**, 591.

FLAKS, A. & LAWS, J. D. (1968) Pulmonary Adenomata Induced by Carcinogen Treatment in Organ Culture. Influence of Duration of Treatment. *Br. J. Cancer*, **22**, 839.

FLAKS, B. & FLAKS, A. (1969) Fine Structure of Murine Pulmonary Adenomata Induced by Carcinogen Treatment in Organ Culture. *Cancer Res.*, **29**, 1781.

FLAKS, B. & FLAKS, A. (1970) Fine Structure of Murine Pulmonary Adenocarcinomata Induced by Treatment with 20-methylcholanthrene in Organ Culture. *Eur. J. Cancer*, **6**, 477.

GOH, S. H. & HARVEY, R. G. (1973) K-Region Arene Oxides of Carcinogenic Aromatic Hydrocarbons. *J. Am. chem. Soc.*, **95**, 242.

GROVER, P. L. (1974) K-Region Epoxides of Polycyclic Hydrocarbons: Formation and Further Metabolism by Rat-lung Preparations. *Biochem. Pharmac.*, **23**, 333.

GROVER, P. L., HEWER, S. & SIMS, P. (1974) Metabolism of Polycyclic Hydrocarbons by Rat-lung Preparations. *Biochem. Pharmac.*, **23**, 323.

GROVER, P. L., SIMS, P., HUBERMAN, E., MARQUARDT, H., KUROKI, T. & HEIDELBERGER, C. (1971) *In vitro* Transformation of Rodent Cells by K-region Derivatives of Polycyclic Hydrocarbons. *Proc. natn. Acad. Sci. U.S.A.*, **68**, 1098.

GROVER, P. L., SIMS, P., MITCHLEY, B. C. V. & ROE, F. J. C. (1975) The Carcinogenicity of Polycyclic Hydrocarbon Epoxides in Newborn Mice. *Br. J. Cancer*, **31**, 182.

HUBERMAN, E., KUROKI, T., MARQUARDT, H., SELKIRK, J. K., HEIDELBERGER, C., GROVER, P. L. & SIMS, P. (1972) Transformation of Hamster Embryo Cells by Epoxides and Other Derivatives of Polycyclic Hydrocarbons. *Cancer Res.*, **32**, 1391.

MARQUARDT, H., KUROKI, T., HUBERMAN, E., SELKIRK, J. K., HEIDELBERGER, C., GROVER, P. L. & SIMS, P. (1972) Malignant Transformation of Cells Derived from Mouse Prostate by Epoxides and Other Derivatives of Polycyclic Hydrocarbons. *Cancer Res.*, **32**, 716.

MARQUARDT, H., KUROKI, T., HUBERMAN, E., SELKIRK, J. K., HEIDELBERGER, C., GROVER, P. L. & SIMS, P. (1974) Malignant Transformation *in vitro* of Mouse Fibroblasts by 7,12-dimethylbenz(a)anthracene and 7-hydroxymethylbenz(a)-anthracene and by their K-region Derivatives. *Int. J. Cancer*, **13**, 304.

MILLER, E. C. & MILLER, J. A. (1967) Low Carcinogenicity of the K-region Epoxides of 7-methylbenz(a)anthracene and Benz(a)anthracene in the

Mouse and Rat. *Proc. Soc. exp. Biol. Med.*, **124**, 915.

NEBERT, D. W. & GELBOIN, H. V. (1969) The *in vivo* and *in vitro* Induction of Aryl Hydrocarbon Hydroxylase in Mammalian Cells of Different Species, Tissues, Strains and Developmental and Hormonal States. *Archs biochim. Biophys.*, **134**, 76.

NEWMAN, M. S. & BLUM, S. (1964) A New Cyclisation Reaction Leading to Epoxides of Aromatic Hydrocarbons. *J. Am. chem. Soc.*, **86**, 5598.

ROE, F. J. C., MITCHLEY, B. C. V. & WALTERS, M. (1963) Tests for Carcinogenesis using Newborn Mice, 1,2-benzanthracene, 2-naphthylamine, 2-naphthylhydroxylamine and Ethyl Methane Sulphonate. *Br. J. Cancer*, **17**, 255.

ROE, F. J. C. & SALAMAN, M. H. (1955) Further Studies on Incomplete Carcinogenesis: Triethylene Melamine, 1,2-benzanthracene and β-propiolactone as Initiators of Skin Tumour Formation in the Mouse. *Br. J. Cancer*, **9**, 177.

SCRIBNER, J. D. (1973) Tumor Initiation by Apparently Noncarcinogenic Polycyclic Aromatic Hydrocarbons. *J. natn. Cancer Inst.*, **50**, 1717.

SIMS, P. (1967) The Carcinogenic Activities in Mice of Compounds Related by 3-methylcholanthrene. *Int. J. Cancer*, **2**, 505.

SIMS, P. (1973) The Preparation and Metabolism of Epoxides Related to 7,12-dimethylbenz(a)-anthracene. *Biochem. J.*, **131**, 405.

SIMS, P. & GROVER, P. L. (1974) Epoxides in Polycyclic Aromatic Hydrocarbon Metabolism and Carcinogenesis. *Adv. Cancer Res.*, **20**, 165.

SIMS, P., GROVER, P. L., SWAISLAND, A. PAL, K. & HEWER, A. (1974) Metabolic Activation of Benzo(a)pyrene Proceeds by a Diol-epoxide. *Nature, Lond.*, **252**, 326.

STEINER, P. E. & EDGECOMBE, J. H. (1952) Carcinogenicity of 1,2-benzanthracene. *Cancer Res.*, **12**, 657.

SVOBODA, D. J. (1962) Ultrastructure of Pulmonary Adenomas in Mice. *Cancer Res.*, **22**, 1197.

SWAISLAND, A. J., GROVER, P. L. & SIMS, P. (1973) Some Properties of "K-region" Epoxides of Polycyclic Aromatic Hydrocarbons. *Biochem. Pharmac.*, **22**, 1547.

SWAISLAND, A. J., HEWER, A., PAL, K., KEYSELL, G. R., BOOTH, J., GROVER, P. L. & SIMS, P. (1974) Polycyclic Hydrocarbon Epoxides: the Involvement of 8,9-dihydro-8,9-dihydroxybenz-(a)anthracene 10,11-oxide in Reactions with the DNA of Benz(a)anthracene-treated Hamster Embryo Cells. *FEBS Letters*, **47**, 34.

TROWELL, O. A. (1954) A Modified Technique for Organ Culture *in vitro*. *Expl cell Res.*, **6**, 246.

VAN DUUREN, B. L., LANGSETH, L., GOLDSCHMIDT, B. M. & ORRIS, L. (1967) Carcinogenicity of Epoxides, Lactones and Peroxy Compounds. VI. Structures and Carcinogenic Activity. *J. natn. Cancer Inst.*, **39**, 1217.